# Anti-VEGF-A Affects the Angiogenic Properties of Tumor-Derived Microparticles

**DOI:** 10.1371/journal.pone.0095983

**Published:** 2014-04-21

**Authors:** Michal Munster, Ella Fremder, Valeria Miller, Neta Ben-Tsedek, Shiri Davidi, Stefan J. Scherer, Yuval Shaked

**Affiliations:** 1 Department of Molecular pharmacology, Rappaport Faculty of Medicine, Technion, Haifa, Israel; 2 Hoffmann La Roche, Basel, Switzerland; European Institute of Oncology, Italy

## Abstract

Tumor derived microparticles (TMPs) have recently been shown to contribute to tumor re-growth partially by inducing the mobilization and tumor homing of specific bone marrow derived pro-angiogenic cells (BMDCs). Since antiangiogenic drugs block proangiogenic BMDC mobilization and tumor homing, we asked whether TMPs from cells exposed to an antiangiogenic drug may affect BMDC activity and trafficking. Here we show that the level of VEGF-A is reduced in TMPs from EMT/6 breast carcinoma cells exposed to the anti-VEGF-A antibody, B20. Consequently, these TMPs exhibit reduced angiogenic potential as evaluated by a Matrigel plug and Boyden chamber assays. Consistently, BMDC mobilization, tumor angiogenesis, microvessel density and BMDC-colonization in growing tumors are reduced in mice inoculated with TMPs from B20-exposed cells as compared to mice inoculated with control TMPs. Collectively, our results suggest that the neutralization of VEGF-A in cultured tumor cells can block TMP-induced BMDC mobilization and colonization of tumors and hence provide another mechanism of action by which antiangiogenic drugs act to inhibit tumor growth and angiogenesis.

## Introduction

Tumors undergo an angiogenic switch when the balance between pro-angiogenic and anti-angiogenic factors is perturbed, leading to tumor outgrowth and expansion [1], [2], [3]. Endothelial cells, which either rapidly divide from pre-existing vessels or home from the circulation to the tumor, actively participate in the tumor angiogenic process [4]. Endothelial progenitor cells (EPCs) constitute the major cell type to incorporate into the blood vessel wall in a systemic angiogenesis process, also called vasculogenesis [5]. In addition, other bone marrow derived cell (BMDC) types, such as myeloid derived suppressor cells (MDSCs), hemangiocytes, and Tie-2 expressing monocytes (TEMs) were also found to contribute to systemic tumor angiogenesis by supporting blood vessel growth and function via different paracrine mechanisms [6].

The contribution of EPCs to tumor blood vessel growth is controversial [7], [8], [9]. We recently demonstrated that the level of EPCs in the peripheral blood of mice rises rapidly in response to various cytotoxic agents, including chemotherapy and vascular disrupting agents (VDAs). Subsequently, these cells home to the treated tumor site, induce angiogenesis and thus aid in tumor cell repopulation leading to tumor re-growth [10], [11]. TEMs and tumor associated macrophages (TAMs) have also been found to colonize treated tumors, and promote revascularization following therapy [12], [13], [14]. Importantly, the addition of an antiangiogenic drug to chemotherapy substantially reduces EPC mobilization and homing to the treated tumor site, leading to enhanced treatment efficacy in part by blocking rebound angiogenesis [10], [11]. Importantly, studies have demonstrated that it is the response of the host, rather than the tumor cells themselves, to such anti-cancer therapies, that facilitates systemic angiogenesis [15], [16].

Tumor cells shed microparticles (MPs) which are a subset of microvesicles (MVs) along with exosomes. MPs vary in size (0.1–1 µm) and primarily contain cell membrane proteins and phospholipids representative of the cells they originate from [17], [18]. Levels of circulating MPs in the blood increase significantly in a variety of disease states, including cancer [19]. Recent findings suggest that tumor-derived MPs (TMPs) may act as messengers and mediators of tumor growth. TMPs containing the oncogenic form of the endothelial growth factor receptor (EGFRvIII) expressed on glioma tumor cells were found to be fused with tumor cells lacking this oncogene [20], [21]. Thus, a new way of communication between tumor cells in the tumor bed or at distant sites could be mediated by TMPs [21]. In a recent study we demonstrated that TMPs from cells exposed to paclitaxel chemotherapy induced BMDC mobilization and colonization of tumors, thereby contributing to angiogenesis and tumor re-growth [22]. However, the impact of antiangiogenic therapy in this context has not been elucidated.

Here we studied the effect of the anti-VEGF-A antibody, B20, on the angiogenic potential of TMPs collected from EMT/6 breast carcinoma cells. We show that the angiogenic properties of TMPs from cells exposed to anti-VEGF-A antibody are reduced due to a reduction in the VEGF-A content, when compared to TMPs from control cells. We demonstrate that TMPs from cells exposed to antiangiogenic therapy do not promote BMDC mobilization and endothelial cell homing to the tumor site. Overall, our results suggest that in addition to the antiangiogenic activity of anti-VEGF-A on endothelial cells, this treatment strategy may also inhibit the angiogenic properties of MPs shed from tumor cells in an anti-VEGF-A microenvironment.

## Materials and Methods

### Cell Culture

EMT-6 and 4T1 murine breast carcinoma and MDA-MB-231 human breast carcinoma cell lines were purchased from the American Type Culture Collection (ATCC, Manassas, VA, USA). Cell lines were grown in Dulbecco’s modified Eagle’s medium (DMEM) supplemented with 10% fetal calf serum, 1% L-glutamine, 1% sodium-pyruvate and 1% streptomycin. Human umbilical vein endothelial cells (HUVECs) (Lonza, Switzerland) were cultured in plates covered with 10% fibronectin (1 mg/ml Biological Industries, Beit HaEmek, Isreal) following 37°C incubation for 30 min. HUVECs were cultured in M199 medium (Sigma-Aldrich, Rehovot, Israel) supplemented with 20% heat inactivated fetal calf serum (FCS), 50 mg/ml endothelial cell growth supplement (ECGS), 50 mg/ml heparin, 10 mM Hepes, 1% L-glutamine, 1% sodium-pyruvate and 1% streptomycin.

### Microparticle Extraction and Quantification

**Figure 1:**
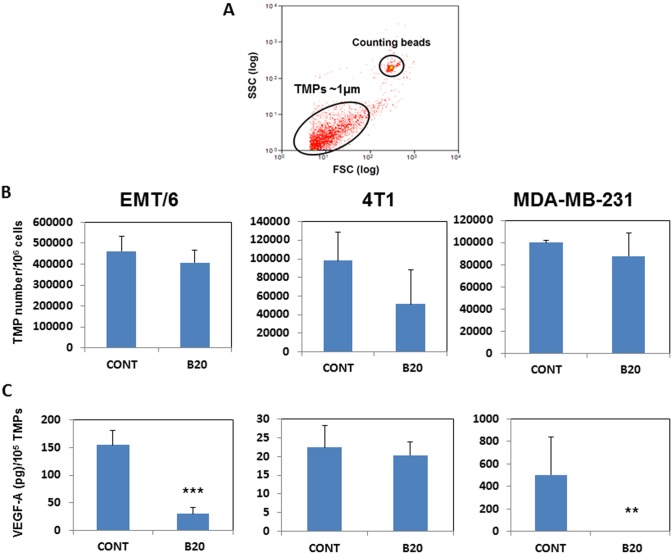
Exposing tumor cells to anti-VEGF-A antibodies reduces the level of VEGF-A in TMPs without affecting the number of TMPs. (A) A representative flow cytometry dotplot for TMP quantification. TMPs are approximately 1 µm, and counting beads are 7.35 µm. The number of TMPs per sample was calculated as the ratio between the number of events collected in the counting beads gate and the number of events collected in the TMPs gate over the total number of counting beads loaded in the sample. (B) EMT/6, 4T1 and MDA-MB231 breast carcinoma cells were either left untreated or exposed to 2 µg/ml B20 antibody for 48 h. TMPs were purified from conditioned medium and quantified by flow cytometry. Shown are the means ± S.D. of triplicates. (C) An equal number of TMPs (100,000) from untreated or B-20-exposed EMT/6, 4T1 and MDA-MB231 breast carcinoma cells were used to quantify the level of VEGF-A by ELISA. In some experiments control for B20 antibodies was used in a form of IgG in culture. Shown are the means ± S.D. of triplicates. **, 0.01>p>0.001.

Cultured cells were grown in medium containing 10% fetal calf serum until they reached 80% confluency, at which point, the medium was replaced with serum free (SF) medium in the presence or absence of 2 µg/ml of B20, an antibody neutralizing both human and murine vascular endothelial growth factor (VEGF-A; kindly provided by Genentech Inc., San Francisco, CA, USA)[23], [24]. In some experiments non-related IgG antibody was used as a control. After 48 hours, conditioned medium was collected and centrifuged at 1500 g/300 g for 20 minutes at 24°C to remove floating cells. The cell free supernatants were then centrifuged at 20,000 g for 1 hour at 4°C. The pellet was then washed with phosphate buffered saline (PBS), and the TMP-containing pellet was resuspended in PBS. TMPs were stored at −80°C until analyzed. Quantification of TMPs was performed using flow cytometry as previously described [22], [25]. Briefly, 0.78 µm-sized beads (Calbiochem) were used to gate on TMP size. TMP number was obtained by calculating the ratio between 7.35 µm counting beads and the number of events collected at the TMP gate. A representative flow cytometry analysis of TMPs is presented in Figure 1A.

### Quantification of the Expression Levels of VEGF-A

Half a million TMPs from MDA-MB231, 4T1 or EMT/6 cell cultures were applied to either human or murine VEGF-A enzyme-linked immunosorbent assay (ELISA) kits (R&D systems) in order to detect VEGF-A levels, in accordance with the manufacturer’s instructions. Experiments were performed in triplicates.

### Invasion and Migration of HUVECs Assessed by the Modified Boyden Chamber Assay

HUVEC invasion and migration properties were assessed using Matrigel- or fibronectin-coated Boyden chambers as previously described [26]. Briefly, serum-starved HUVEC cells (2×10^5^ cells per 0.2 ml medium) were added to the upper chamber that was coated with 50 µl Matrigel (BD Biosciences, San Jose, CA, USA) for assessing invasion, or with 50 µl fibronectin (10 µg/ml) for assessing migration. The lower compartment was filled with PBS that contained 5×10^6^ TMPs from control cells or from cells exposed to 2 µg/ml B20 antibody, and subsequently lysed by 4 repeated freeze-thaw cycles. After 4 hours for migration, or 24 hours for invasion, HUVECs which migrated to the lower compartment of the chamber, were stained with crystal violet and images were captured using the Leica CTR 6000 microscope system (Leica Microsystems, Wetzlar, Germany) followed by cell counting. The experiments were carried out in triplicate, and results presented as means ± SD.

### Microvessel Sprouting using Aortic Ring

One millimeter long aortic rings (n = 3/group) obtained from 8–10 week old BALB/c mice, were embedded in Matrigel (BD Bioscience) and overlaid with SF DMEM supplemented with 0.1×10^6^ TMPs from control cells or from cells exposed to B20 antibodies. Plates were incubated at 37°C in a humidified 5% CO^2^ atmosphere and the medium was replaced every other day. Images of the rings and microvessel sprouting were captured using Leica CTR 6000 microscope (Leica Microsystems).

### Matrigel Plug Assay

Matrigel (0.5 ml; BD Biosciences) that contained TMPs from control cells or from cells exposed to B20 antibodies, was injected subcutaneously into each flank of 8–10 week old BALB/c mice (n = 4 mice/group). Plugs were removed 10 days later, and subsequently prepared for either histological assessment using hematoxylin and eosin (H&E) and endothelial cell staining, or flow cytometry for the evaluation of BMDCs. For flow cytometry, Matrigel plugs were prepared as a single cell suspension as previously described [27] and cells that infiltrated the plugs were identified as described below.

### Animals and Tumor Model

Half a million EMT/6 cells were implanted subcutaneously in the flanks of 8–10 week old BALB/c mice (Harlan Biotech Israel, Rehovot, Israel). Tumor size was assessed regularly with Vernier calipers using the formula, width^2^×length×0.5. Mice were intravenously injected twice weekly with 0.5×10^6^ TMPs collected from control cells or from cells exposed to B20 antibodies. Control mice were injected with PBS. Tumors were removed at end point (∼1000–1500 mm^3^). All animal studies and experimental protocols were approved by the Animal Care and Use Committee of the Technion.

### Evaluation of BMDCs by Flow Cytometry

BMDCs obtained from tumors, Matrigel plugs following single cell suspension, or whole blood following red blood cell lysis were analyzed by flow cytometry using the following antibody mixtures: CXCR4+/CD11+/VEGFR1+/CD45+ for identifying hemangiocytes; CD11b+/Gr-1+ for identifying MDSCs; and VEGFR2+/7AAD/CD117+/CD45- for identifying viable CEPs as previously described [28]. All monoclonal antibodies were purchased from BD Biosciences, R&D systems, and Macs Militenyi Biotec (Bergisch Gladbach, Germany), and used according to the manufacturers’ instructions. At least 100,000 events were acquired using a CyAn ADP flow cytometer and analyzed with Summit software (Beckman Coulter, Nyon, Switzerland).

### Tissue Processing and Immunostaining

Tumors were embedded in OCT (Tissue-Tek, Sakura Finetek USA Inc., USA) and stored at −80°C. Matrigel plugs were embedded in 10% paraformaldehyde at room temperature (RT) for 24 hours. Subsequently, the plugs were embedded in OCT at 4°C for 48 hours and then stored at −80°C. Tumors or Matrigel plugs were cryosectioned (4–6 µm and 20–25 µm respectively), and then immunostained with an endothelial cell specific antibody (anti-mouse CD31, 1∶200, BD Biosciences) and a secondary antibody conjugated with Cy3 (1∶150, Jackson ImmunoResearch Laboratories Inc., West Grove, PA, USA), and with a pan-hematopoietic marker (anti-mouse CD45 conjugated with FITC, 1∶150, BD Biosciences). Tumor cryosections were also used for analysis of blood vessel perfusion by Hoechst 33342 (40 mg/kg) (Sigma-Aldrich Israel Ltd., Rehovot, Israel), injected to mice 90 sec before mice were sacrificed, as previously described [10]. The number of vessel structures (positive for CD31 staining) and/or functional vessels (positive for Hoechst and CD31 staining) per field were counted and plotted (approximately 5 fields per tumor, n>20 fields/group). Tumor and Matrigel plugs sections were visualized under a Leica CTR 6000 microscope system (Lieca Microsystems).

### Statistical Analysis

Data are presented as means ± standard deviation (SD). Statistically significant differences in mean values were assessed by one-way ANOVA, followed by Newman-Keuls ad hoc statistical test using GraphPad Prism 4 software (La Jolla, CA, USA). When applicable, statistical significance comparing only two groups was determined by two-tailed Student t-test. Significance was set at values of *P<.05, **P<.01, and ***P<.001.

## Results

### The Angiogenic Content but not Number of TMPs is Altered following anti-VEGF-A Drug Therapy

To investigate the effect of the anti-VEGF-A B20 antibody on TMPs, EMT/6, 4T1 and MDA-MB-231 breast carcinoma cells were exposed to B20 antibodies for 48 hours or left untreated. TMPs were then purified from the conditioned medium and quantified by flow cytometry as described [22], [29]. A representative flow cytometry analysis of TMPs is shown in Figure 1A. The numbers of TMPs derived from the three cell lines treated with B20 antibodies were not significantly different to those from untreated control cells (Figure 1B). However, the VEGF-A content was substantially reduced in TMPs derived from EMT/6 and MDA-MB-231 but not 4T1 B20-exposed cells as compared to controls (Figure 1C).

### TMPs from Cells Exposed to an Antiangiogenic Drug are Unable to Activate Endothelial Cells

**Figure 2:**
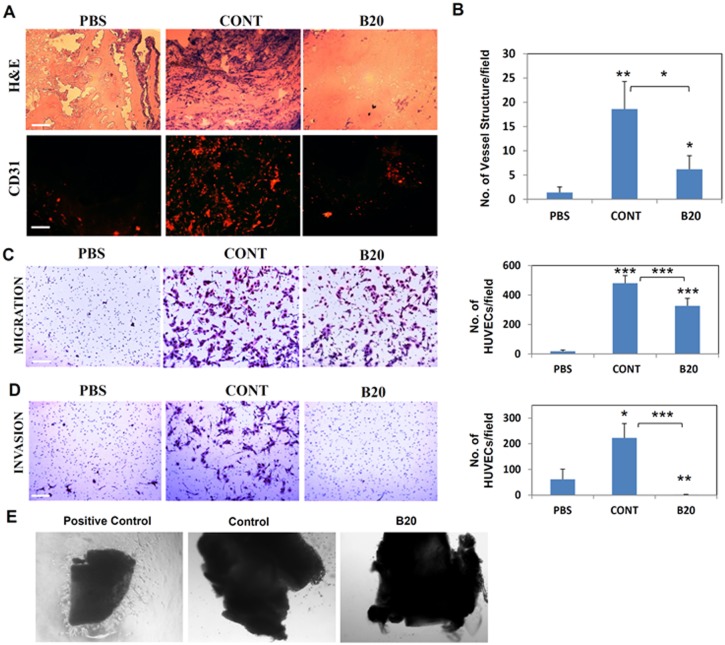
TMPs from cells exposed to anti-VEGF-A antibody exhibit reduced ability to promote endothelial cell activity. Matrigel plugs containing an equal number of TMPs (0.5×10^6^) from untreated or B20-exposed EMT/6 cells were implanted into the flanks of 8–10 week old BALB/c mice. Matrigel plugs containing PBS were used as a negative control. Ten days later, plugs were removed and then sectioned. (A) Slides were stained with H&E or immunostained with the endothelial cell marker CD31 (designated in red) (scale bar = 100 µm). (B) Microvessel density in the plugs was calculated by counting vessel structures. (C–D) An equal number of TMPs (5×10^6^) from untreated or B20-exposed EMT/6 cells were tested for HUVEC migration (C) and invasion (D) using the modified Boyden chamber assay. PBS was used as a negative control. Cells invading the membrane of the Boyden chamber were stained with Crystal Violet and images were captured using a Leica CTR 6000 microscope. The number of cells invading the membrane were counted and plotted (n>8/group). (E) Aortic rings from BALB/c mice (n = 4/group) were cultured in medium containing 0.1×106 TMPs from untreated or B20-exposed EMT/6 cells. Endothelial cell medium (ECGS) was used as a positive control. Images were captured using an inverted light microscope system (Leica CTR 6000 system) (Scale bar = 500 µm). *, 0.05<p<0.01; **, 0.01>p>0.001; ***, p<0.001.

To analyze the angiogenic properties of TMPs from cells exposed to B20 antibodies, equal numbers of TMPs derived from B20-exposed or control untreated EMT/6 cells were mixed with Matrigel and implanted in mice. As a negative control, mice were implanted with Matrigel containing PBS. After ten days, plugs were removed and stained with H&E or anti-CD31 antibodies to assess host cell colonization and angiogenesis. As expected, the number of colonizing host cells, among those endothelial cells, was greater in plugs containing TMPs from control cells as compared to control-PBS plugs, consistent with a previous study [22]. However, the number of host cells and endothelial cells (in red) in plugs containing TMPs from B20-exposed cells was significantly lower than their numbers in plugs containing TMPs from control cells (Figure 2A). In addition, microvessel density was significantly lower in the Matrigel plugs containing TMPs from cells exposed to B20 antibody when compared to plugs containing TMPs from control cells (p<0.05, Figure 2B).

Next, we evaluated the migration and invasion properties of endothelial cells in the presence of TMPs collected from untreated control or B20-exposed cells. Medium without added TMPs was used as a negative control. The medium containing TMPs from control cells induced invasion and migration of HUVECs through the Boyden chamber, similarly to a previous study [22], whereas medium containing TMPs from B20-exposed cells induced significantly lower numbers of migrating and invading HUVECs (p<0.01, Figure 2C and 2D). In addition, angiogenic activity, determined by microvessel sprouting in murine aortic rings, was not detected in the presence of TMPs collected from B20-exposed cells in contrast to control TMPs (Figure 2E). These results suggest that TMPs promote vessel sprouting and angiogenesis only when they originate from control cells; once tumor cells are exposed to anti-VEGF-A antibodies, their TMPs lose the ability to promote endothelial cell activity.

### TMPs Derived from anti-VEGF-A-treated Cells Alter BMDC Mobilization

**Figure 3:**
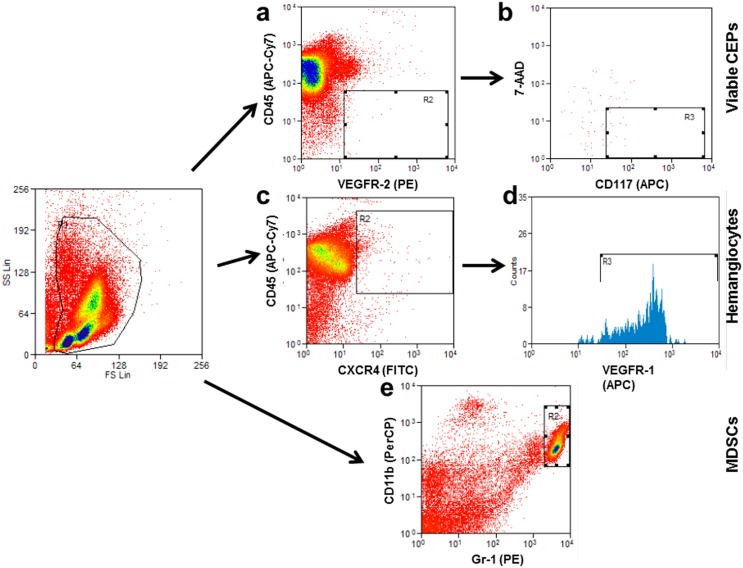
Representative flow cytometry plots of viable CEPs, hemangiocytes, and myeloid derived suppressor cells. An example of the analysis of flow cytometry data obtained from peripheral blood of BALB/c mice is presented. Viable CEPs are determined as (a) positive for VEGFR2 and negative for CD45 as well as (b) positive for CD117 and negative for 7-AAD. Hemangiocytes are determined as (c) positive for CD45 and CXCR4 as well as (d) positive for VEGFR1. Myeloid derived suppressor cells (MDSCs) are determined as positive for (e) both Gr-1 and CD11b.

**Figure 4:**
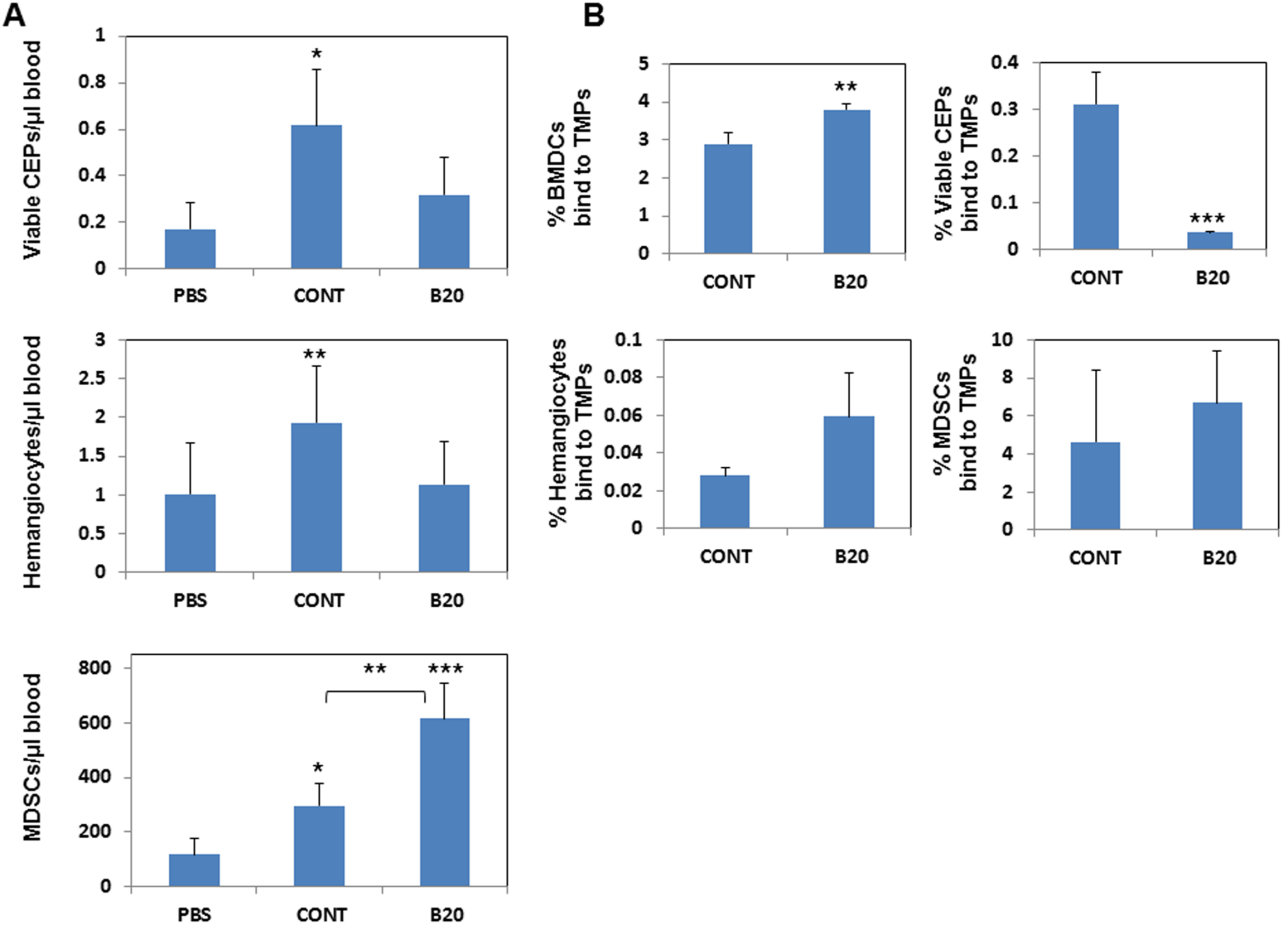
TMPs from cells exposed to anti-VEGF-A antibody do not induce viable CEP and hemangiocyte mobilization. (A) An equal number of TMPs (0.5×10^6^) from untreated (CONT) or B20-exposed EMT/6 cells was injected into the tail vein of 8–10 week old non-tumor bearing BALB/c mice (n = 4 mice/group). Control mice were injected with PBS (PBS). One hour later, blood was drawn from the retro-orbital sinus for the evaluation of viable CEPs (CD45−/VEGFR2+/CD117+/7AAD−), MDSCs (Gr1+/CD11b+), and hemangiocytes (CD11b+/CXCR4+/VEGFR1+) using flow cytometry. (B) Half a million TMPs from untreated (CONT) or B20-exposed cells were tagged with PKH26, and subsequently injected into the tail vein of BALB/c mice (n = 4 mice/group). Control mice were injected with PBS. One hour later, blood was drawn by cardiac puncture and total BMDCs (CD45+), viable CEPs, hemagiocytes, and MDSCs were analyzed by flow cytometry. The percentage of the different cell types positive for tagged TMPs was plotted. **, 0.01>p>0.001; ***, p<0.001.

BMDCs, such as viable CEPs, hemangiocytes, and MDSCs, are known to promote tumor angiogenesis [30]. Therefore, to further understand the role of TMPs in angiogenesis, we investigated whether TMPs play a role in BMDC mobilization as well as the effect of anti-VEGF-A therapy on this process. To this end, half a million TMPs collected from EMT/6 cells exposed to B20 antibody or their control counterparts were injected into the tail vein of 8–10 week old BALB/c mice. Mice injected with PBS were used as a negative control. One hour later, blood was drawn from the retro-orbital sinus and the levels of viable CEPs, hemangiocytes, and MDSCs were analyzed by flow cytometry. A representative flow cytometry analysis of the different BMDC types is shown in Figure 3. The results shown in Figure 4A demonstrate that the mobilization of all BMDC populations was substantially increased in mice injected with control TMPs as compared to PBS-injected mice, consistent with a previous study [22]. However, the mobilization of viable CEPs and hemangiocytes was not significantly different in mice injected with TMPs derived from B20-exposed and control cells although reduced viable CEP and hemangiocyte mobilization was observed. Interestingly, significantly higher levels of MDSCs were induced upon injection of TMPs from B20-exposed cells as compared to control cells (Figure 4A). It should be noted that the number of MDSCs colonizing tumors was found to substantially increase in antiangiogenic-treated tumors [31]. Overall, these results suggest that TMPs from B20-exposed cells do not affect systemic angiogenesis induced by viable CEPs and hemangiocyes, yet it can promote MDSC mobilization.

To assess whether TMPs bind directly to BMDCs and whether this binding is affected by anti-VEGF-A therapy, TMPs from control or B20-exposed cells were tagged with PKH26, a fluorescent dye which binds to membrane lipids, and incubated for one hour with BMDCs obtained from the femurs of BALB/c mice. One hour later, the BMDCs were immunostained for viable CEPs, hemangiocytes and MDSCs and subsequently analyzed by flow cytometry. TMPs from control and B20-exposed cells bound to hemangiocytes and MDSCs to similar extents. Binding of TMPs from B20-exposed cells to BMDCs was slightly but significantly increased, whereas binding to viable CEPs was dramatically decreased as compared to control TMPs (Figure 4B). Overall, these results suggest that TMPs bind directly to several types of BMDCs; however, once they are derived from B20-exposed cells, their binding properties to specific BMDCs are altered.

### TMPs Derived from anti-VEGF-A-treated Cells do not Promote BMDC Colonization of Matrigel Plugs or Tumors

**Figure 5:**
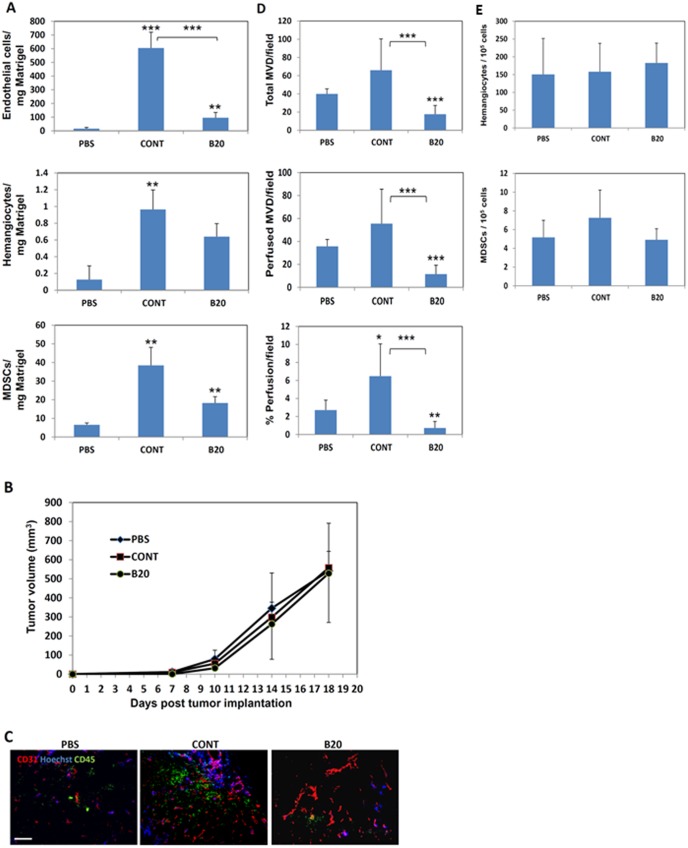
TMPs from cells exposed anti-VEGF-A antibody do not promote angiogenesis in tumors. (A) Matrigel plugs containing an equal number of TMPs (0.5×10^6^) from untreated or B20-exposed EMT/6 cells were implanted into the flanks of 8–10 week old BALB/c mice. Matrigel plugs containing PBS were used as a negative control. Ten days later, plugs were removed and prepared as single cell suspensions. The extracted cells were immunostained for endothelial cells, hemangiocytes and MDSCs and analyzed by flow cytometry. Results are presented as the number of cells per 1 mg Matrigel. (B–E) Eight to ten week old BALB/c mice (n = 4 mice/group) were implanted with 0.5×10^6^ EMT/6 cells into the flanks. When tumors reached a size of approximately 50 mm^3^, injections with 0.5×10^6^ TMPs from untreated or B20-exposed EMT/6 cells were performed twice weekly. Control mice were injected with PBS. (B) Tumor growth was assessed by a Vernier caliper using the formula, width^2^×length×0.5. Tumors were removed at endpoint, and subsequently were either (C) stained for CD31 (in red), CD45 (in green), and Hoechst (in blue) for the evaluation of (D) microvessel density and perfusion (scale bar = 100 µm), or (E) prepared as single cell suspensions for the evaluation of MDSCs and hemangiocytes colonization of tumors using flow cytometry. **, 0.01>p>0.001; ***, p<0.001.

We next compared the profile of BMDCs that colonize Matrigel plugs in the presence of TMPs collected from B20-exposed and control EMT/6 cells. To this end, equal numbers of TMPs derived from B20-exposed or control untreated EMT/6 cells were mixed with Matrigel and implanted in mice. As a negative control, mice were implanted with Matrigel containing PBS. After ten days, plugs were removed, prepared as single cell suspensions and analyzed by flow cytometry for the presence of endothelial cells, hemangiocytes, and MDSCs. The number of all these cell types was lower in Matrigel plugs containing TMPs from B20-exposed cells when compared to control TMPs, although the number of hemangiocytes and MDSCs did not reach statistical significance. Of note, as previously demonstrated [22], Matrigel plugs containing PBS revealed minimal or no colonization of any of the BMDC types tested (Figure 5A). We next asked whether TMP-induced BMDC colonization has any effect on tumor growth. To test this, mice bearing EMT/6 tumors were injected twice weekly through the tail vein with 0.4×10^6^ TMPs purified from control or B20-exposed EMT/6 cell cultures. PBS injections were used as a negative control. Tumor volumes were assessed by a caliper, and tumors were removed at endpoint in order to evaluate angiogenesis and BMDC colonization. Interestingly, no significant changes in tumor volumes and growth were observed between the groups (Figure 5B). However, significant increases in microvessel density, functional vessels, and percentage of perfusion were observed in tumors of mice injected with TMPs from control cells compared with TMPs from B20-exposed cells or PBS control (Figure 5C and 5D). Again, no differences in hemangiocyte and MDSC colonization of tumors were observed between the groups (Figure 5E). Collectively, these results indicate that extrinsic addition of TMPs does not affect tumor growth, at least not at this tumor stage, but does alter the angiogenic properties in such tumors. TMPs from control cells exhibit a higher angiogenic potential than TMPs from cells treated with an antiangiogenic drug.

## Discussion

Tumor cell repopulation and regrowth is often observed during the therapy-break periods between successive acute chemotherapies [15], [16]. Our previous studies demonstrated that the induction of BMDC-mediated angiogenesis, particularly CEPs, can contribute to tumor re-growth, and it is partially mediated by SDF-1 and G-CSF [11], [16], [32]. Since some of these experiments were conducted in non-tumor bearing mice, we suggested that the host response to chemotherapy promotes angiogenesis therefore contributing to tumor re-growth [33]. In a subsequent study, we focused on the contribution of tumor cells to angiogenesis. TMPs from breast carcinoma cells exposed to paclitaxel chemotherapy induced BMDC mobilization and tumor homing, a process which was partially regulated by osteopontin [22]. Thus, chemotherapy affects tumor re-growth by two different processes. On the one hand, it stimulates production of various cytokines and growth factors in the host which in turn promote BMDC mobilization and tumor homing [32], and on the other hand, it promotes the production of TMPs from tumor cells which then can contribute to the same process [22]. It should be noted that increased number of TMPs has also been found in breast cancer patients undergo chemotherapy treatment [22], suggesting that the effects we observed in our in vitro tumor model could be recapitulated in vivo. In the current study, we report that the tumor proangiogenic effects induced by TMPs can be blocked by an antiangiogenic drug with the focus on anti-VEGF-A therapy. We show that TMPs from cells exposed to an anti-VEGF-A antibody have a reduced ability to stimulate BMDC mobilization and subsequent colonization of tumors.

The number of TMPs has been shown to substantially increased when tumor cells were exposed to paclitaxel chemotherapy when compared to untreated tumor cells [22]. In contrast, in the current study, we show that exposing cells to anti-VEGF-A neutralizing antibodies did not result in a significant change in the number of TMPs. The differences between the two scenarios could be related to the fact that tumor cells undergo apoptosis in the presence of cytotoxic chemotherapy drug, while these apoptotic effects were absent when cells were exposed to a cytostatic drug which inhibits endothelial cells, and not tumor cells. We also demonstrated that TMPs from both untreated and B20-exposed cells bound directly to BMDCs with similar binding affinities, although the TMPs from cells exposed to anti-VEGF-A antibodies exhibited reduced angiogenic potential compared to TMPs from control cells by means of reduced HUVEC migration and invasion as well as BMDC colonization in Matrigel plugs. Consistently with the in vitro findings, also mice inoculated with TMPs from B20-exposed cells exhibited reduced microvessel density, functional vessels, and percentage of perfusion in tumors compared to mice inoculated with control TMPs. Thus TMPs from cells exposed to anti-VEGF-A antibodies inhibit the angiogenesis activities in growing tumors. It is yet to be determined whether other anti-angiogenic drugs such as small molecule tyrosine kinase inhibitors, e.g., sorfenib or sunitinib, would act in the same manner on TMPs by means of reducing their angiogenic content. It should be noted that the antiangiogenic content in microvessicles have been previously studied. For example, exosomes released from retinal astroglial cells possess antiangiogenic content. Therefore, they inhibit the activity of neovascularization in the eye, hence promoting its protection from vascular dysfunction (such as age-related macular degeneration) [34]. Interestingly, in our study we demonstrated that tumor volume was not significantly different between the mice inoculated with TMPs from B20-exposed cells when compared to mice inoculated with TMPs from untreated control cells. Although the reasons for these findings were not uncovered, it is plausible that in a different tumor model in which tumor cells do not divide so rapidly, changes in tumor growth could have been observed, or when tumors would have left to grow for additional period of time.

The presence of B20 antibodies in culture altered the expression levels of VEGF-A and the angiogenic properties of TMPs. As such, it can explain the inhibition of viable CEP, hemangiocyte, and MDSC mobilization although the latter two did not reach statistical significance. Previous studies indicated that various proteins are enriched in MPs compared to their cell of origin [35], [36]. As such, VEGF, similarly to other proangiogenic and antiangiogenic factors, may be enriched in TMPs in the same manner. In a previous study, TMPs extracted from paclitaxel-exposed cells resulted in changes of various cytokines and growth factors as analyzed by a protein array. Among them, we found that SDF-1 was upregulated in TMPs from paclitaxel-exposed cells when compared to control TMPs [22]. Although in this study we solely focused on VEGF-A as we used an anti-VEGF-A antibody, it would be of interest to identify whether other pro-angiogenic and anti-angiogenic factors are affected by the lack of VEGF-A. Studying the properties of TMPs requires a more refined proteomic analysis. Nonetheless, we can speculate that the lack of VEGF-A in TMPs from cells exposed to anti-VEGF therapy could be due to the fact that B20 antibody interferes with the autocrine loop of VEGF-A in tumor cells [37]. Another possibility for the antiangiogenic activity of TMPs from cells exposed to anti-VEGF-A antibody could be due to the uptake of B20 antibodies either by tumor cells or by their TMPs, as recently was suggested that platelets can uptake bevacizumab, the humanized antibody against VEGF-A [38], and therefore it is plausible that such antibodies will be present in MPs. It should be noted that TMPs were undergo vigorous washes in order to minimize traces of B20 antibodies in the culture. Overall, these results indicate that B20 may affect the tumor microenvironment not only via direct antiangiogenic activity on endothelial cells but also through the inhibition of specific BMDC colonization of tumors thereby preventing tumor systemic angiogenesis.

One of the major current obstacles in clinical oncology, especially in the case of antiangiogenic therapy, is the lack of suitable and reliable biomarkers to predict clinical outcome [39]. Several clinical and preclinical biomarkers such as levels of circulating endothelial cells in the peripheral blood, SNP-analysis of genes related to angiogenesis, among others, have been suggested (for review see [39]). More recently, the angiogenic profile of cancer stem cells (CSCs) has been shown preclinically to correlate with antiangiogenic treatment outcomes [40]. In this study, exposing cells to B20 antibodies resulted in reduced levels of VEGF-A in TMPs, suggesting that MPs should be further investigated as a surrogate biomarker for antiangiogenic activity. Indeed, MP-based technology is currently being tested in search of potential prognostic or predictive biomarkers for tumor growth [20]. It has been shown that platelets can take up bevacizumab [38], and as such platelets, as well as MPs found in plasma of bevacizumab-treated patients may serve as a biomarker for antiangiogenic therapy. In this regard, levels of MPs and their content in cancer patients have already been studied as diagnostic and prognostic biomarkers. For example, circulating levels of endothelial MPs and leukocyte MPs were found to correlate with CEA and CA15-3 both of which are breast cancer biomarkers [17]. Furthermore, levels of plasma TMPs were elevated in patients with progressed gastric cancer [41]. As such, MPs could serve as potential diagnostic and/or prognostic biomarkers in the clinical settings. However, there are a few challenges in the isolation and purification of MPs as there are no standardized protocols for MPs’ extraction and evaluation. In addition, MPs vary in size and type, and a greater distinction between the different populations of MPs is required, by using methods that are likely to provide accurate sizing of MPs compared with conventional flow cytometry [29]. Therefore, in the context of this study it would be of interest to evaluate levels of VEGF-A in TMPs or MPs, and correlate them with the clinical outcome of antiangiogenic drug treatments, with the notion that extensive efforts should be made to translate these pre-clinical results into standardized clinical testing.
